# Retinitis pigmentosa with pre-mRNA processing factor 31 mutation: a case report of a Moroccan patient

**DOI:** 10.11604/pamj.2025.51.77.47184

**Published:** 2025-07-22

**Authors:** Mariyam Khallouqi, Rachid El Jaoudi, Hassan Ghazal, Bahia Chahdi Ouazzani

**Affiliations:** 1 Laboratory of Medical Biotechnology, Faculty of Medicine and Pharmacy, Mohammed V University, Rabat, Morocco,; 2 Laboratory of Precision Medicine and One Health, School of Medicine, Mohammed VI University of Sciences and Health, Casablanca, Morocco,; 3 Department of Sports Sciences, Laboratory of Sports Sciences and Performance Optimization, Royal Institute of Executive Management in Youth and Sport, Salé, Morocco,; 4 National Center for Scientific and Technical Research, Rabat, Morocco,; 5 Faculty of Medicine and Pharmacy, Mohammed V University, Rabat, Morocco

**Keywords:** Retinitis pigmentosa, next generation sequencing, PRPF31, night blindness, case report

## Abstract

To report the clinical findings of a Moroccan patient presenting with retinitis pigmentosa (RP), together with pre-mRNA processing factor 31 (PRPF31) mutations. The patient experienced night blindness a few months before being diagnosed with RP in July 2023, and several ophthalmic examinations were carried out, including best-corrected visual acuity (BCVA) measurement, slit-lamp biomicroscopy, dilated ophthalmoscopy, fundus photography, optical coherence tomography (OCT), and electroretinogram (ERG). A genetic analysis was carried out to identify the causative genes in order to better understand the disease and facilitate the path to therapy. The variant found was in the PRPF31 gene at position c.1165C>T p(Gln389**), generating a stop codon and a loss of 110 amino acids. This mutation has previously been described as pathogenic and induces autosomal dominant RP. This is the first description of a Moroccan case presenting RP with a PRPF31 mutation. More cases are needed to better define the clinical progression and the range of mutations.

## Introduction

Rod-cone dystrophies, also called retinitis pigmentosa (RP), are the leading cause of blindness or visual impairment in the young adult population, with an estimated prevalence of one in 3500 individuals and more than one million people affected worldwide [1]. RP is indeed a clinically and genetically heterogeneous group of inherited retinal disorders; it encompasses a wide range of clinical presentations and is caused by various genetic mutations. Clinically, the rod photoreceptors and retinal pigment epithelial (RPE) cells are usually the first cells to degenerate, followed by secondary degeneration of cone photoreceptors. When the cone dysfunctions occur, progressive visual field constriction and loss of central vision may be observed. A very severe form of visual impairment could be attended, even blindness, with no effective treatment so far [2]. Genetically, mutations in over 80 genes have been identified, and nearly 3100 mutations have been reported to date. These genes are involved in various functions within the photoreceptors (rods and cones) and other retinal cells. RP is also characterized by diversification of inheritance patterns, including dominant inheritance mode, recessive, X-linked, and digenic inheritance modes [3].

Diagnosing RP involves a combination of clinical evaluation, imaging, and genetic testing. Funduscopic examination shows in RP patients bone spicule retinal pigmentation, chorioretinal atrophy, attenuated retinal vessels, and waxy pallor optic disc. Also, ERG shows a severely reduced or nondetectable response reflecting impaired photoreceptor function [4]. In RP patients, autofluorescence shows clearly the retinal degeneration and pigment accumulation. Given the genetic and phenotypic diversity, genetic testing is crucial for diagnosing RP and identifying the specific mutation involved. This can also help in understanding the prognosis and exploring potential treatments [4]. In this study, we report a case of a patient with RP and a PRPF31 mutation on whole-exome sequencing.

## Patient and observation

**Figure 1:**
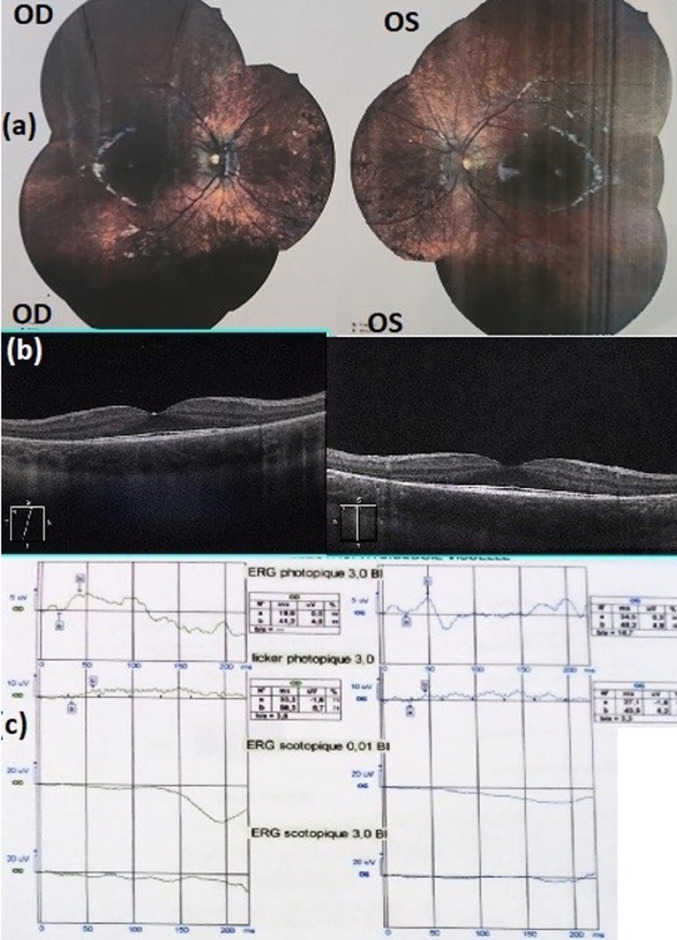
fundus finding of the patient (a), optical coherence tomography (b), and electroretinogram (c)

**Patient information:** the patient, an 8-year-old Moroccan girl, showed symptoms a few months before being diagnosed with RP in July 2023. During the follow-up, several ophthalmic examinations were carried out, including BCVA measurement, slit-lamp biomicroscopy, dilated ophthalmoscopy, fundus photography, OCT, and ERG. Since she was examined at the University Hospital Center Avicenna- specialty hospital, to be part of this study (Figure 1). The color fundus photography taken during the initial visit revealed retinal degeneration characterized by peripheral bone-spicule equatorial pigmentation, while the retinal vessels and optic disc remained within normal limits. OCT indicated preserved inner retinal layers but demonstrated retinal atrophy in the central macular region. Additionally, the OCT section showed an interruption of the outer nuclear layers in the perivascular region, suggesting a loss of photoreceptors. No evidence of macular edema was observed. The ERG indicated a significant disorder of retinal electrogenesis, particularly affecting the rods.

**Clinical assessment:** the exposed case was detected as part of a genetic study carried out at the Faculty of Medicine and Pharmacy of Rabat on Moroccan patients who suffer from retinitis pigmentosa. This study has the approval of the ethics committee for biomedical research (CERB: 63/21 deliberated on October 23, 2023).

**Figure 2:**
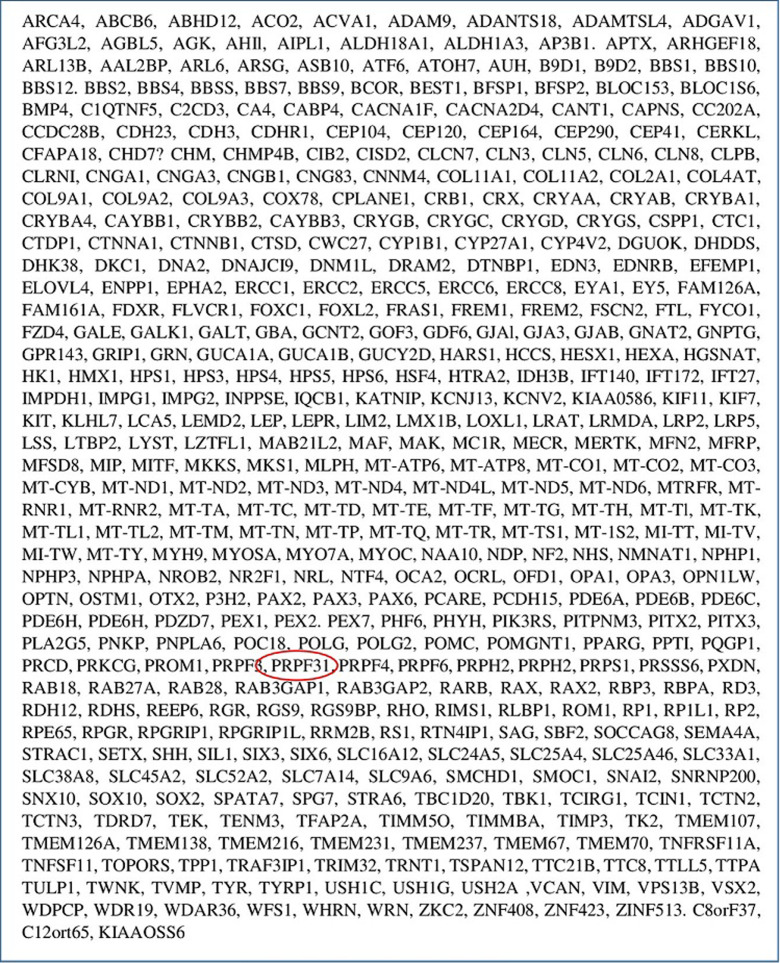
gene panel including PRPPF31 (mutated gene)

**Genetic analyses:** the patient has been followed for a year, and a genetic test was suggested for a better understanding of the disease. Genomic analysis was performed using next-generation sequencing (NGS-based CNV analyses). The Deoxyribonucleic Acid (DNA) was enzymatically fragmented, and regions of interest were enriched using DNA capture probes. The final indexed libraries are sequenced on an Illumina platform. The gene panel used (Figure 2) included the gene mutated in PERPF31. The nucleotide coverage was 99.67%.

A heterozygous likely pathogenic variant was identified in the PRPF31 gene (Table 1). The PRPF31 c.1165C>T p(Gln389*) creates a premature stop codon and loss of 110 amino acids (PRPF31 gene generates a protein with 499 amino acids). According to the Human Gene Mutation Database (HGMD) professional, this variant has previously been described as causing retinal dystrophy by Ezquerral-inchausti (PMID: 30337596). It is classified as likely pathogenic (class 2).

**Table 1:** description of the sequence variants

Gene	Variant coordinates	Amino-Acid change	SNP identifier	Zygosity	In SILICO parameters	Allele frequencies	Type and classification
PRPF31	NM_015629.3: c.1165C>T	p.(Gln389*)	N/A	Heterozygous	PolyPhen:- Align-GVDG:N/A MutatioNTASTER:Disease causing Conservtion_nt:hignt Conservation_aa:N/A	GnomAD: ESP:1000G CentoMD	Nonsense Likely pathogenic (class 2)

**Clinical finding:** the patient began showing visual symptoms such as night blindness a year ago, and she was diagnosed in May 2023. Her ophthalmological work-up showed a visual acuity of OD: +2.5 (2,175') 9/10, OG: +2.75 (2,5,175') 9/10. An OCT and ERG were performed to assess the state of the retina. The OCT showed retinal atrophy beyond the central macular region. The OCT slice showed an interruption of the outer nuclear layer in the perimacular region, indicating a loss of photoreceptors. The inner layers were preserved. The ERG exposed severely diminished photopic responses and extinguished scotopic responses, indicating a major disturbance in retinal electrogenesis, especially rods. Fundus examination revealed diffuse retinal degeneration, together with peripheral bone-spicule pigmentation, vessels, and the optic disc was within normal limits. She had no relevant medical history, used no medicines, and was not exposed to toxins or illicit drugs.

**Patient's perspectives:** the patient, an 8-year-old child diagnosed with retinitis pigmentosa due to a PRPF31 mutation, reports difficulties with vision, particularly in low light. The child and his family have noted problems with night vision, making it difficult to navigate dark spaces and engage in certain activities. The patient has expressed frustration at not being able to see as clearly as her peers, particularly during play and school activities. Despite these challenges, the child is adapting with the help of assistive devices such as magnifiers and special glasses. The family is actively involved in helping the patient adjust to his condition, and the patient remains optimistic about future treatments. The family has expressed hope that advances in research will improve the patient's condition and provide a better quality of life in the future.

**Informed consent:** the patient's tutors were informed about the study procedure, then a consent form was provided and fully signed.

## Discussion

The prevalence of RP is indeed around 1-3 in every 5.000 people, making it relatively rare but significant for those affected. It can be caused by mutations in several genes and different transmission routes, each of which may contribute to the disease in various ways [1]. In the gene panel analyzed, a mutation in the PRPF31 gene was found. This gene is specifically linked to autosomal dominant retinitis pigmentosa-11 (OMIM #606419) [4], which is compatible with this case. The patient is heterozygous for the Gln389Ter mutation, which was already described in the ClinVar database [5] with a pathogenic/likely pathogenic classification. Mutations in the pre-mRNA processing factors (PRPFs) are described as the second most common cause of autosomal dominant RP (adRP) after mutations in rhodopsin [6]. Among these factors, mutations in PRPF31 are the most common, with a prevalence ranging from 5% to 8% in different adRP cohorts across various geographical regions [7]. The PRPF31 gene encodes a protein that is part of the spliceosome, a complex machinery responsible for splicing pre-mRNA into mature mRNA. This splicing process is crucial for proper gene expression [8]. Deficiency of PRPF31 leads to a retina-specific phenotype. PRPF31 mutations induce significant morphological and functional changes in RPE cells and photoreceptor cells, notably an altered cell morphology with loss of typical cellular architecture, and disruption of the cellular monolayer integrity [9]. Also, retinal organoids derived from patient-specific induced pluripotent stem cells (iPSCs) with PRPF31 mutations show that rod photoreceptors are typically the first to undergo degeneration. This mirrors the clinical manifestation of night blindness and loss of peripheral vision in RP patients [10]. Following the initial loss of rod cells, cone photoreceptors often begin to deteriorate, inducing impairment of central vision [10].

The patient presents a clinical form similar to the phenotype of RP with mutated PRPF31. The destabilization of retinal cell structure elucidated in the literature is represented in this case by retinal atrophy beyond the central macular region. Also, the loss of photoreceptors was detected by OCT, showing an interruption of the outer nuclear layer in the perimacular region. The crucial clue to the PRP31 mutation demonstrated in the study of patient-specific induced pluripotent stem cell (iPSC)-derived retinal organoids [10] was also found in this patient, namely the loss of rod photoreceptors followed by a loss of cone exposed by the patient's ERG findings of severely diminished photopic responses and extinguished scotopic responses, indicating a major disruption of retinal electrogenesis, particularly of the rods.

Following the classification of the PRPF31 c.1165C>T p(Gln389*) mutation as pathogenic/likely pathogenic for autosomal dominant retinitis pigmentosa-11 and the clinical presentation of the patient, which was more or less similar to the phenotype described in the literature, we can conclude that this phenotype could be due to this variant. However, this study can only be complete and concrete if it is extended to a cohort of patients with the same clinical manifestations and the same mutation, followed by a study of the mutation in vitro for a better phenotype-genotype correlation.

**Limitations:** although this study provides valuable insights into this case of retinitis pigmentosa, it is important to be aware of several limitations that may affect the interpretation of the results, since to correlate a genotype to phenotype, an extension of the cohort must be elaborated and a functional study of the mutation in vitro is necessary. These limitations provide directions for future research and should be taken into account when applying the results to different contexts.

## Conclusion

In this study, the description of the clinical case in favor of retinitis pigmentosa has also been concretized by the description of the variant that may be at the origin of the disease in this patient, although we will not be able to correlate the genotype associated with the PRPF31 mutation with the phenotype until we extend the study to a large cohort of patients with the same mutation and the same symptoms, and until we concretize the effect of the loss of 110 AA of the PRPF31 protein *in vitro*.

## References

[1] Hartong DT, Berson EL, Dryja TP (2006). Retinitis pigmentosa. Lancet.

[2] Verbakel SK, van Huet RAC, Boon CJF, den Hollander AI, Collin RWJ, Klaver CCW (2018). Non-syndromic retinitis pigmentosa. Prog Retin Eye Res.

[3] Pan X, Chen X, Liu X, Gao X, Kang X, Xu Q (2014). Mutation analysis of pre-mRNA splicing genes in Chinese families with retinitis pigmentosa. Mol Vis.

[4] Vilela MA, Menna Barreto RK, Menna Barreto PK, Sallum JM, Mattevi VS (2018). Novel codon 15 RHO gene mutation associated with retinitis pigmentosa. Int Med Case Rep J.

[5] Deery EC, Vithana EN, Newbold RJ, Gallon VA, Bhattacharya SS, Warren MJ (2002). Disease mechanism for retinitis pigmentosa (RP11) caused by mutations in the splicing factor gene PRPF31. Hum Mol Genet.

[6] National Center for Biotechnology Information ClinVar

[7] Audo I, Bujakowska K, Mohand-Saïd S, Lancelot ME, Moskova-Doumanova V, Waseem NH (2010). Prevalence and novelty of PRPF31 mutations in French autosomal dominant rod-cone dystrophy patients and a review of published reports. BMC Med Genet.

[8] Tanackovic G, Ransijn A, Thibault P, Abou Elela S, Klinck R, Berson EL (2011). PRPF mutations are associated with generalized defects in spliceosome formation and pre-mRNA splicing in patients with retinitis pigmentosa. Hum Mol Genet.

[9] Waseem NH, Vaclavik V, Webster A, Jenkins SA, Bird AC, Bhattacharya SS (2007). Mutations in the gene coding for the pre-mRNA splicing factor, PRPF31, in patients with autosomal dominant retinitis pigmentosa. Invest Ophthalmol Vis Sci.

[10] Rodrigues A, Slembrouck-Brec A, Nanteau C, Terray A, Tymoshenko Y, Zagar Y (2022). Modeling PRPF31 retinitis pigmentosa using retinal pigment epithelium and organoids combined with gene augmentation rescue. NPJ Regen Med.

